# Relation between low-density lipoprotein cholesterol/apolipoprotein B ratio and triglyceride-rich lipoproteins in patients with coronary artery disease and type 2 diabetes mellitus: a cross-sectional study

**DOI:** 10.1186/s12933-017-0606-7

**Published:** 2017-10-02

**Authors:** Shigemasa Tani, Tsukasa Yagi, Wataru Atsumi, Kenji Kawauchi, Rei Matsuo, Atsushi Hirayama

**Affiliations:** 1 Department of Health Planning Center, Nihon University Hospital, 1-6 Kanda-Surugadai, Chiyoda-ku, Tokyo, 101-8309 Japan; 2 Department of Cardiology, Nihon University Hospital, Tokyo, Japan; 3 Division of Cardiology, Department of Medicine, Nihon University School of Medicine, Tokyo, Japan

**Keywords:** Coronary artery disease, Diabetes mellitus, LDL-particle size, Triglyceride-rich lipoproteins

## Abstract

**Background:**

The low-density lipoprotein cholesterol/apolipoprotein B (LDL-C/apoB) ratio has conventionally been used as an index of the LDL-particle size. Smaller LDL-particle size is associated with triglyceride (TG) metabolism disorders, often leading to atherogenesis. We investigated the association between the LDL-C/apoB ratio and TG metabolism in coronary artery disease (CAD) patients with diabetes mellitus (DM).

**Methods:**

In the cross-sectional study, the LDL-C/apoB ratio, which provides an estimate of the LDL-particle size, was calculated in 684 consecutive patients with one additional risk factor. The patients were classified into 4 groups based on the presence or absence of CAD and DM, as follows: CAD (−) DM (−) group, n = 416; CAD (−) DM (+) group, n = 118; CAD (+) DM (−) group, n = 90; CAD (+) DM (+) group, n = 60.

**Results:**

A multi-logistic regression analysis after adjustments for coronary risk factors revealed that the CAD (+) DM (+) condition was an independent predictor of the smallest LDL-C/apoB ratio among the four groups. Furthermore, multivariate regression analyses identified elevated TG-rich lipoprotein (TRL)-related markers (TG, very-LDL fraction, remnant-like particle cholesterol, apolipoprotein C-II, and apolipoprotein C-III) as being independently predictive of a smaller LDL-particle size in both the overall subject population and a subset of patients with a serum LDL-C level < 100 mg/dL. In the 445 patients followed up for at least 6 months, multi-logistic regression analyses identified increased levels of TRL-related markers as being independently predictive of a decreased LDL-C/apoB ratio, which is indicative of smaller LDL-particle size.

**Conclusions:**

The association between disorders of TG metabolism and LDL heterogeneity may account for the risk of CAD in patients with DM. Combined evaluation of TRL-related markers and the LDL-C/apoB ratio may be of increasing importance in the risk stratification of CAD patients with DM. Further studies are needed to investigate the useful clinical indices and outcomes of these patients.

*Clinical Trial Registration* UMIN (http://www.umin.ac.jp/) Study ID: UMIN000028029 retrospectively registered 1 July 2017

## Introduction

Progression of coronary atherosclerosis in patients with diabetes mellitus (DM) is characterized by increased frequency, extent, complexity, and rate of progress compared to that in non-DM patients [1–3].

In a meta-analysis that assessed the preventive effect of statins on cardiovascular (CV) events, the preventive effect on CV mortality was found to be no more than 20% even when the serum low-density lipoprotein cholesterol (LDL-C) level was controlled with a statin [4], and among the residual risks of statin therapy, insulin resistance, Impaired glucose tolerance, and lipid metabolism abnormalities [5], especially defective triglyceride (TG) metabolism, was found to cause a decrease in LDL-particle size, which has a powerful atherogenic effect [6].

Moreover, TG metabolites, i.e., chylomicrons, very low density lipoprotein (VLDL), and remnant-like particle cholesterol (RLP-C), which are TG-rich lipoproteins (TRLs), and, apolipoprotein (apo) C-II and apo C-III which are involved in the metabolic process, etc., have been demonstrated to be involved in the progression of atherosclerosis [6].

Density gradient ultracentrifugation, nondenaturing gradient gel electrophoresis, and nuclear magnetic resonance spectroscopy are the methods that are usually employed to measure LDL-particle diameter; however, these methods present problems in clinical settings due to their cost and complexity [7]. Each LDL-particle has one apolipoprotein (apo) B molecule, which is recognized by LDL receptors that clear LDL from the plasma. Thus, the apoB concentration represents the plasma number of LDL-particles. Thus, the LDL-C/apoB ratio reflects indirectly the LDL-particle size [7].

An LDL-C/apoB ratio of 1.2 has been suggested to correspond to an LDL diameter of 25.5 nm, which is the cut-off value for distinguishing LDL pattern A (large buoyant LDL) from LDL pattern B [small dense (sd)-LDL] [7, 8], indicating the presence of a large quantity of sd-LDL. In fact, the Québec Cardiovascular Study demonstrated that in patients with LDL-particle sizes of 25.5 nm or smaller, the CAD incidence increased significantly as the serum LDL-C level increased, while in patients having large LDL-particle sizes of 26.0 nm or greater, no significant difference in CAD events was observed according to the absolute serum LDL-C level [9].

We hypothesized that reduction of the LDL-C/apoB ratio is associated with disordered TG metabolism, particularly with increase of TRLs, and that the LDL-C/apoB ratio is lower in CAD patients with underlying DM than in CAD patients without DM, patients with DM alone and patients without CAD or DM.

The purpose of this study was to evaluate the LDL-C/apoB ratio as a marker of LDL-particle size in CAD patients with DM in a hospital-based cross-sectional study, and to clarify the relationships between the LDL-C/apoB ratio and TRL-related markers (TG, VLDL, RLP-C,apo C-II, and apo C-III), which are indicators of TG metabolism.

## Methods

### Study design and populations

This study was designed as a hospital-based cross-sectional study to investigate the relationship between the LDL-C/apoB ratio and TRL-related markers including TG, VLDL, RLP-C, apoC-II, and apo C-III, in CAD patients with type-2 diabetes. In addition, we examined the relationships among the changes in the LDL-C/apoB ratio and changes in the TRL-related markers in those cases that were still available for additional measurements 6 months later. This study is the sub-analysis of our previous study, which showed LDL-particle size and TG-metabolism disorder using cross-sectional method [10].

The study was conducted on a sample of 700 consecutive outpatients with the presence of one or more risk factors for CAD, who had undergone regular examinations for treatment of their various diseases at the Cardiovascular Center, Nihon University Surugadai Hospital between April 2009 and October 2009. This study was conducted in accordance with the ethical principles of the Declaration of Helsinki. The study protocol was approved by the Institutional Review Board at our institute and written informed consent was obtained from all the study participants.

The criterion for patient registration in the cross sectional study was the presence of one or more risk factors for CAD. The diagnostic criteria for the coronary risk factors used in this study were as follows; hypertension: systolic pressure of ≥ 140 mmHg and/or a diastolic pressure of ≥ 90 mmHg, or taking antihypertensive medication; diabetes mellitus: fasting plasma glucose ≥ 126 mg/dL and HbA1c ≥ 6.5%, or current treatment with anti-diabetic agents; lipid disorder: serum LDL-C ≥ 140 mg/dL, serum TG ≥ 150 mg/dL, and/or serum high-density lipoprotein cholesterol (HDL-C) less than 40 mg/dL, or current treatment with lipid-modifying medication; Cigarette smoking was defined as current smoking or smoking cessation within 1 year prior to the start of the study; chronic kidney disease: Estimated glomerular filtration rate (eGFR) < 60 ml/min/1.73 m^2^; obesity: body mass index ≥ 25 kg/m^2^. A diagnosis of hyperuricemia was made when the serum uric acid level was 7.0 mg/dL or above, or taking medications.

Angiographically, CAD was defined as a history of documented myocardial infarction, prior coronary revascularization intervention (coronary artery bypass graft surgery or percutaneous coronary intervention), or the presence of ≥ 50% stenosis in 1 or more of the coronary arteries identified during cardiac catheterization.

Patients were not enrolled if they met any of the following exclusion criteria: hepatic dysfunction (alanine aminotransferase and aspartate aminotransferase ≥ 2 times the upper limit of the normal values), known malignant disease, or diagnosis of acute coronary syndrome within 3 months prior to the study.

### Measurement of laboratory parameters

Fasting blood samples were collected early in the morning after a 12-h fast. The serum total cholesterol (TC), HDL-C, and TG levels were measured by the standard methods. The serum LDL-C level was estimated by using the Friedewald formula [11]. The VLDL fraction was measured by performing polyacrylamide-gel electrophoresis using the LipoPhor system (Joko, Tokyo, Japan). The RLP-C level was measured by an immunoadsorption assay (SRL Inc., Tokyo, Japan). The serum apolipoproteins were determined by turbidimetric latex agglutination assays (SRL). The malondialdehyde-modified LDL (MDA-LDL) level was measured by an enzyme-linked immunosorbent assay (SRL). The high sensitivity C-reactive protein (hs-CRP) level was measured by a nephelometric assay (Behring Diagnostic Marburg, Germany). The eGFR was calculated by using the abbreviated MDRD (Modification of Diet in Renal Disease) study formula modified by a Japanese coefficient [12].

### Statistical analysis

Data are expressed as the mean ± standard deviation (SD) for continuous variables and as percentages for discreet variables. Data that did not have a normal distribution were expressed as medians (interquartile range). The data for categorical variables were analyzed by the χ^2^ test. For the subset analysis of four groups according to the presence or absence of DM and CAD, we used analysis of variance (ANOVA) followed by Bonferroni’s correction for covariates if differences were found in the patient characteristics or laboratory profile markers. In this study, an LDL-C/apoB ratio of 1.2 was deemed to correspond to an LDL particle size of 25.5 nm (the cutoff sd-LDL particle size), consistent with previous reports [7–9], and an LDL-C/apoB ratio of < 1.2 served as a dependent variable for the multivariate logistic regression analysis described below. A detailed multi-logistic analysis of each group was performed in which the presence or absence of CAD and DM was evaluated in patients with an LDL-C/apoB ratio of less than 1.2. Accordingly, multi-logistic regression analyses were performed with no adjustments (model 1), after adjustment for age and gender (model 2), and after adjustment for traditional coronary risk factors and concomitant use of drugs with an action that increases LDL-particle size (e.g., statins, fibrates, and glitazones [13]) (model 3). These analyses were used to evaluate the association between LDL-C/apoB ratios of less than 1.2 and the prevalence of CAD or DM. Furthermore, univariate and multivariate regression analyses were performed to identify independent variables of LDL-C/apoB ratio. As the TRLs-related markers constituted mutually confounding factors, five multivariate regression models incorporating the respective variables were established to carry out the analyses. All variables correlated with LDL-C/apoB ratio at p < 0.05 in the univariate regression analysis were entered into the multivariate model. For the cases that were still available for additional measurements 6 months later, a multi-logistic regression analysis was performed to identify variables that were significantly associated with the changes in the LDL-C/apoB ratio. Increase/decrease of the LDL-C/apoB ratio from the baseline was entered as a dependent variable, and the age, gender, CAD risk factors, and the absolute changes of the TRL-related markers were entered as independent variables. We used SPSS Window ver 12.0 (Statistical Package for the Social Sciences, SPSS Ins., Chicago, IL) for all analyses.

## Results

### Subjects

We excluded 16 subjects from the study because of missing laboratory data. A final total of 684 subjects were included in the study. The participants consisted of 470 (59%) male and 214 (41%) female patients. The patients were classified into the four groups according to the presence or absence of CAD and/or DM. Comparison of the four groups according to the presence or absence of CAD and/or DM is shown in Tables 1, 2, and 3.

**Table 1:** Patient characteristics

Variables	All cases n = 684	CAD (+)			CAD (+)			p value among the 4 groups
		DM (+) n = 60	DM (−) n = 90	p value between the 2 groups	DM (+) n = 118	DM (−) n = 416	p value between the 2 groups	
Male/female, n (%)	470 (69)/214 (31)	54 (90)/6 (10)	79 (88)/11 (12)	0.674	83 (70)/35 (30)	254 (61)/162 (39)	0.732	< 0.0001
Age (years)	62 ± 14	65 ± 10.3	64 ± 11.3	0.529	68 ± 113	60 ± 16	< 0.0001	< 0.0001
BMI (kg/m2)	24.1 ± 3.9	24.6 ± 3.5	24.4 ± 3.8	0.810	24.7 ± 4.1	23.7 ± 4.0	0.001	0.050
BMI ≥ 30, n (%)	51 (7.5)	4 (6.7)	5 (5.6)	0.911	13 (11.3)	30 (7.2)	0.049	0.384
Hypertension, n (%)	479 (70)	51 (85)	66 (73)	0.078	85 (72)	279 (67)	0.302	0.027
Cigarette smoking, n (%)	103 (15)	8 (13)	9 (10)	0.544	18 (15)	67 (16)	0.939	0.554
Dyslipidemia, n (%)	458 (67)	60 (100)	71 (82)	0.0005	107 (91)	216 (52)	< 0.0001	< 0.0001
Hyperuricemia	130 (19)	13 (25)	20 (22)	0.589	21 (18)	71 (17)	0.670	0.423
eGFR (ml/min/1.73m2)	70.4 ± 18.5	68.8 ± 14.0	69.0 ± 16.0	0.278	72.6 ± 20.4	70.3 ± 19.1	0.307	0.467
CKD stage 3 ≥, n (%)	192 (28)	15 (25)	28 (31)	0.401	30 (25)	116 (28)	0.562	0.697
CAD, n (%)	150 (22)						–	–
Effort AP/OMI	46/104	17 (28)/43 (78)	29 (32)/62 (68)	0.613	–	–	–	–
BNP (pg/mL)	23.1 (11.1/46.3)	32.5 (15.2/53.2)	35.5 (13.7/71.0)^2^	0.407	28.9 (15.1/75.4)^1^	19.4 (9.8/38.6)	0.081	0.0162
Cerebral Infarction, n (%)	20 (2.9)	2 (3.3)	3 (3.3)	0.705	8 (6.8)	7(1.7)	0.003	0.036
Peripheral arterial disease, n (%)	10 (1.5)	2 (3.3)	2 (2.2)	0.696	5 (4.2)	1 (0.2)	0.0005	0.006
HbA1c (%)	5.94 ± 0.77	6.83 ± 1.12^3,4^	5.70 ± 0.34	< 0.0001	6.68 ± 0.86^3,4^	5.64 ± 0.42	< 0.0001	< 0.0001
Duration of diabetes (years)*	–	5.3 (3.3/9.8)	–	6.2 (4.3/7.5)–	–	–		
Concomitant drugs (%)								
Antiplatelets	190 (27.8)	55 (92)	69 (77)	0.017	60 (25)	37 (8.9)	< 0.0001	< 0.0001
ACE inhibitors	52 (7.6)	11 (18)	14 (16)	0.655	6 (5.1)	21 (5.0)	0.770	< 0.0001
ARBs	271 (40)	22 (37)	30 (33)	0.674	54 (46)	165 (40)	0.129	0.312
β Blockers	144 (21)	25 (42)	21 (23)	0.017	27 (23)	71 (17)	0.070	0.0002
Calcium channel blockers	316 (46)	32 (53)	46 (51)	0.790	54 (46)	184 (44)	0.974	0.430
Statins	318 (46)	53 (88)	66 (73)	0.026	59 (50)	140 (34)	0.002	< 0.0001
Fibrates	13 (1.9)	10 (1.7)	1 (1.1)	0.771	7 (5.9)	4 (1.0)	0.002	0.006
Sulfonylurea	48 (7.0)	24 (40)	–	–	23(19)	–	–	–
Metformin	19 (2.8)	8 (10)	–	–	–12 (10)	–	–	
α-Glucosidase inhibitor	45 (6.6)	20 (33)	–	–	23 (19)	–	–	–
Thiazolidine	21 (3.1)	11 (18)	–	–	8 (6.8)	–	–	–
Insulin	4 (0.7)	1 (1.7)	–	–	3 (2.5)	–	–	–

ANOVA and post hoc tests with Bonferroni correction were performed to test between-group differences

*BMI* body mass index, *eGFR* estimated glomelular flow rate; *CKD* chronic kidney disease, *CAD* coronary artery disease, *AP* angina pectoris, *OMI* old myocardial infarction, *Hb* hemoglobin, *ACE* angiotensin-converting enzyme, *ARB* angiotensin receptor blocker

* *Median* interquartile range in parentheses

^1^p < 0.05, ^2^ p < 0.01, ^3^ p < 0.0001 vs. CAD (−) DM (−) group

^4^p < 0.0001 vs. CAD (+) DM (−) group

**Table 2:** Laboratory profile

Variables	All cases n = 684	CAD (+)			CAD (−)			p value among the 4 groups
		DM (+) n = 60	DM (−) n = 90	p value between the 2 groups	DM (+) n = 118	DM (−) n = 416	p value between the 2 groups	
Lipids								
TC (mg/dL)	195 ± 38	176 ± 36^4,7^	179 ± 31^4,7^	0.380	196 ± 36	201 ± 38	0.064	< 0.0001
LDL-C (mg/dL)	109 ± 31	97 ± 27^4,5^	97 ± 25^4,5^	0.710	109 ± 29	114 ± 31	0.209	< 0.0001
HDL-C (mg/dL)	58 ± 17	51 ± 11^4,9^	56 ± 15	0.012	56 ± 18	59 ± 17	0.029	0.0004
non-HDL-C (mg/dL)	138 ± 35	126 ± 34^3,6^	123 ± 28^4,7^	0.866	140 ± 36	142 ± 35	0.363	< 0.0001
TRLs-related markers								
TG (mg/dL)*	122 (88/186)	192 (106/210)^1^	125 (86/173)^5^	0.128	145 (109/221)^3^	115 (83/176)	< 0.0001	0.003
VLDL fraction (%)	12.9 ± 6.6	14.7 ± 6.9^2^	13.5 ± 6.9^1,5^	0.341	15.3 ± 6.9^4^	11.9 ± 6.2	< 0.0001	< 0.0001
RLP-C (mg/dL)*	5.4 (4.0/8.0)	5.3 (3.5/8.7)	4.9 (3.5/7.1)	0.271	6.0 (4.4/9.4)	5.3 (4.0/7.7)	0.027	0.096
apo B (mg/dL)	90 ± 22	85 ± 211	83 ± 17^3,6^	0.640	92 ± 21	92 ± 22	0.943	0.0012
apo C-II (mg/dL)	4.6 ± 2.1	5.2 ± 2.6^2,8^	4.3 ± 1.9^6^	0.044	5.1 ± 2.4^2^	4.4 ± 1.9	0.005	0.0009
apo C-III (mg/dL)	10.2 ± 3.8	10.6 ± 4.6	9.9 ± 3.7	0.303	10.8 ± 4.4	10.0 ± 3.0	0.094	0.108
LDL oxidation marker								
MDA-LDL (U/L)	110 ± 46	106 ± 40	93 ± 33^3,6^	0.051	114 ± 45	114 ± 49	0.207	0.001
Lipid ratio								
TG/HDL-C*	2.32 (1.44/3.60)	2.74 (1.93/4.46)^1^	2.29 (1.66/3.44)^5^	0.032	2.80 (1.84/4.58)^2^	2.04 (1.28/3.34)	0.018	0.007
Inflammatory marker								
WBC count (mm^−3^)	6096 ± 1630	6354 ± 1374	6126 ± 1271	0.258	6391 ± 1745	5960 ± 1685	0.013	0.037
hs-CRP (mg/L)*	0.50 (0.30/1.20)	0.55 (0.22/1.78)	0.40 (0.20/0.90)	0.290	0.80 (0.40/1.60)	0.50 (0.20/1.10)	< 0.0001	0.322

ANOVA and post hoc tests with Bonferroni correction were performed to test between-group differences

*TC* total cholesterol, *LDL* low-density lipoprotein, *HDL* high-density lipoprotein, *TG* triglyceride, *VLDL* very LDL, *RLP* remnant-like particle, apo apolipoprotein, *MDA* malondealdehyde-modified, *WBC* white blood count, *hs-CRP* high-sensitivity C-reactive protein

* *Median* interquartile range in parentheses

^1^p < 0.05, ^2^ p < 0.01, ^3 ^p < 0.001, ^4 ^p < 0.0001 vs. CAD (−) DM (−) group

^5^p < 0.05, ^6 ^p < 0.01, ^7 ^p < 0.001 vs. CAD (−) DM (+) group

^8^p < 0.05 vs. CAD (+) DM (−) group

**Table 3:** Lipid profile

Variables	CAD (+) n = 140	CAD (−) n = 534	p value between the 2 groups	DM (+) n = 190	DM (−) n = 494	p value between the 2 groups
non-HDL-C (mg/dL)	123 ± 31	142 ± 3 5	< 0.0001	139 ± 35	134 ± 35	0.105
TG (mg/dL)*	128 (92/186)	120 (87/187)	0.295	139 (107/218)	119 (89/176)	< 0.0001
VLDL (%)	14.0 ± 6.9	12.6 ± 6.5	0.025	14.8 ± 6.8	12.2 ± 6.3	< 0.0001
RLP-C (mg/dL)*	5.0 (3.5/7.4)	5.4 (4.1/8.0)	0.130	5.7 (4.2/8.9)	5.2 (3.9/7.4)	0.0026
apo C-II (mg/dL)	4.7 ± 2.2	4.6 ± 2.1	0.627	4.7 (3.5/6.1)	4.2 (3.1/5.3)	0.0004
apo C-III (mg/dL)	10.2 ± 4.0	10.2 ± 3.7	0.856	9.7 (8.0/12.3)	9.4 (7.8/11.4)	0.059
LDL-C/apoB ratio*	1.174 (1.073/1.231)	1.241 (1.138/1.333)	< 0.0001	1.173 ± 0.152	1.227 ± 0.152	< 0.0001
TG/HDL-C ratio*	2.497 (1.715/3.824)	2.555 (1.360/3.593)	0.063	3.558 ± 2.648	2.828 ± 2.717	0.002

*TC* total cholesterol, *LDL* low-density lipoprotein, *HDL* high-density lipoprotein, *TG* triglyceride, *VLDL* very LDL, *RLP* remnant-like particle, apo apolipoprotein, *MDA* malondealdehyde-modified, *WBC* white blood count, *hs-CRP* high-sensitivity C-reactive protein

* Median; interquartile range in parentheses

Table 3 shows the results of analysis from another viewpoint to provide a clearer understanding of the features of the lipid profiles for the four categories of patients shown in Table 2. The serum non-HDL-C level was significantly lower in the CAD (+) group than in the CAD (−) group, probably reflecting the statin treatment that is given to many patients of the CAD (+) group. The VLDL fraction was significantly higher in the CAD (+) group than in the CAD (−) group, probably reflecting a higher percentage of patients potentially having abnormal TG metabolism in the CAD (+) group, although the serum TG level did not differ significantly between the two groups. On the other hand, comparison between the DM (+) group and DM (−) group revealed a significantly greater number of patients with high levels of TRL-related markers in the DM (+) group than in the DM (−) group. Furthermore, the LDL-C/apoB ratio was significantly lower in the CAD (+) group than in the CAD (−) group, and TG/HDL-C ratio was higher in the CAD (+) group than in the CAD (−) group, although this difference was not statistically significant. Comparison of the DM (+) and DM (−) groups revealed a significantly lower LDL-C/apoB ratio and significantly higher TG/HDL-C ratio in the DM (+) group than in the DM (−) group.

### Comparison of LDL-C/apoB ratio among the 4 groups

**Fig. 1:**
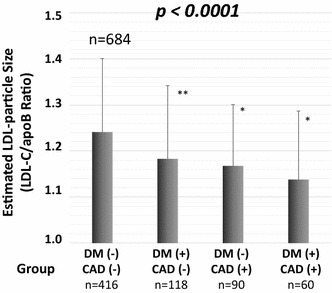
Comparison of LDL-C/apoB ratio among the 4 groups. *DM* diabetes mellitus, *CAD* coronary artery disease;* error bar* mean ± standard deviation; *p < 0.0001, vs. CAD (−) DM(−) group; **p < 0.001 vs. CAD (−) DM(−) group

The LDL-C/apoB ratios of the entire group of patients ranged from 0.622 to 1.694 (mean ± SD: 1.223 ± 0.146, and the LDL-C/apoB ratio ranges according to group were: CAD (+) DM (+) group, 0.622–1.408 (1.153 ± 0.133); CAD (+) DM (−) group, 0.868–1.458 (1.171 ± 0.129); CAD (−) DM (+) group, 0.779–1.525 (1.203 ± 0.124); and CAD (−) DM (−) group, 0.692–1.694 (1.250 ± 0.150)). There were significant differences in the LDL-C/apoB ratios among the 4 groups (p < 0.0001) (Fig. 1).

### Multi-logistic regression analysis to determine the relationship between the LDL-C/apoB ratio corresponding to sd-LDL and the presence or absence of CAD and DM

**Fig. 2:**
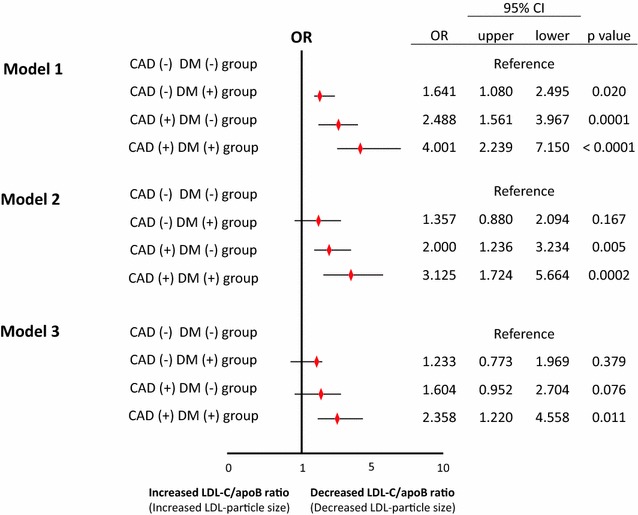
Multi-logistic regression analysis to determine the relationship between LDL-C/apoB ratio corresponding to sd-LDL and the presence or absence of DM and CAD. *DM* diabetes mellitus, *CAD* coronary artery disease, *OR* odds ratio, *CI* confidence interval. CAD (−) DM (−) group, n = 416; CAD (−) DM (+) group, n = 118; CAD (+) DM (−) group, n = 90; DM (+) CAD (+) group, n = 60. For this logistic regression analysis, an LDL-C/apoB ratio of 1.2 was deemed to correspond to an LDL particle size of 25.5 nm (the cutoff sd-LDL particle size) [9, 13, 14], and an LDL-C/apoB ratio of < 1.2 served as a dependent variable. Model 1: Unadjusted. Model 2: Adjusted for age and sex. Model 3: Adjusted for age, sex, hypertension (yes/no), cigarette smoking (yes/no), body mass index, statins use (yes/no), fibratses use (yes/no), and glitazones use, (yes/no)

Multi-logistic regression analyses with no adjustments (model 1), after adjustments for age and gender (model 2), and after adjustments for coronary risk factors and concomitant use of drugs (model 3) were performed to evaluate the association between an LDL-C/apoB ratio of < 1.2 and the prevalence of CAD or DM. The analysis with adjustments for traditional coronary risk factors and concomitant drug use revealed that the CAD (+) DM (+) group was the only group exhibiting a significant and independent variable for a LDL-C/apoB ratio of less than 1.2, both in the overall cohort (Fig. 2), and in the subgroup of patients with serum LDL-C levels of less than 100 mg/dL (data not shown).

### Univariate and multivariate regression analyses of variables identified LDL-C/apoB ratio

All of the variables that correlated with the LDL-C/apoB ratios at p < 0.05 in the univariate regression analysis were entered into the 5 multivariate models. The results of the analyses of all of the multivariate regression models showed that serum TRLs-related markers were significant variables that were independent of LDL-C/apoB ratios. Next, similar analyses were performed in patients with serum LDL-C levels of < 100 mg/dL, patients with serum LDL-C levels of < 100 mg/dL, and patients taking/not taking lipid-modifying treatments. All the univariate and multivariate regression analyses showed that the high levels of TRL-related markers were independent determinants of a low LDL-C/apoB ratio (Table 4). Table 5 shows the correlations between LDL-C/apoB ratio and TRL-related markers among the 4 groups. LDL-C/apoB ratio and serum RLP-C level in the CAD (+) DM (−) group was the only correlation not showing statistical significant; on the other hand, statistically significantly negative correlations were noted between the LDL-C/apoB ratio and all TRL-related markers was noted.

**Table 4:** Univariate and multivariate regression analyses of variables identified LDL-C/apoB ratio

Variables	Univariate		Multivariate 1		Multivariate 2		Multivariate 3		Multivariate 4		Multivariate 5	
	r	p value	β	p value	β	p value	β	p value	β	p value	β	p value
All cases (n = 684)												
Age	−0.151	< 0.0001	−0.104	0.008	−0.125	0.001	−0.134	0.001	−0.144	0.0002	−0.141	0.0001
Gender	−0.196	< 0.0001	−0.135	0.0003	−0.119	0.0005	−0.131	0.0007	−0.113	0.003	−0.079	0.027
BMI	−0.134	0.001	0.020	0.622	0.002	0.960	0.011	0.797	0.018	0.646	0.048	0.201
Cigarette smoking	−0.054	0.163										
Hypertension	−0.154	< 0.0001	−0.014	0.707	−0.009	0.814	−0.019	0.630	−0.024	0.527	−0.008	0.822
Dyslipidemia	−0.244	< 0.0001	−0.047	0.311	−0.082	−0.068	−0.084	0.087	−0.052	0.266	−0.010	0.819
Diabetes mellitus	−0.150	< 0.0001	0.009	0.856	0.027	0.582	<0.0001	0.994	0.004	0.932	−0.009	0.849
HbA1c	−0.131	0.0008	0.01	0.836	0.025	0.612	0.025	0.630	0.032	0.522	0.029	0.542
HDL-C	0.299	< 0.0001	0.155	0.007	0.045	0.295	0.181	< 0.0001	0.236	< 0.0001	0.344	< 0.0001
Statins use	−0.220	< 0.0001	−0.131	0.003	−0.109	0.010	−0.138	0.003	−0.108	0.014	−0.147	0.0004
Fibrates use	−0.122	0.002	−0.075	0.037	−0.059	0.088	−0.081	0.029	0.069	0.052	−0.063	0.061
Glitazone use	−0.035	0.368										
TRLs-related markers												
TG*	−*0.446*	< *0.0001*	−*0.345*	< *0.0001*	–		–		–		–	
VLDL	−*0.502*	< *0.0001*	–		−*0.424*	< *0.0001*	–		–		–	
RLP-C*	−*0.304*	*0.001*	–		–		−*0.240*	< *0.0001*	–		–	
apo C-II	−*0.353*	< *0.0001*	–		–		–		−*0.323*	< *0.0001*		
apo C-III	−*0.401*	< *0.0001*	–		–		–		–		−*0.045*	< *0.0001*
Inflammatory markers												
WBC count	−0.130	0.0007	−0.036	0.339	−0.031	0.387	−0.053	0.170	−0.065	0.082	−0.035	0.318
hs-CRP*	−0.110	0.005	0.002	0.956	−0.018	0.636	0.008	0.843	0.014	0.720	0.026	0.472
LDL-C < 100 mg.dL (n = 264)												
Age	−0.095	0.124										
Gender	0.183	0.003	−0.049	0.358	−0.052	0.318	−0.058	0.290	−0.040	0.437	−0.007	0.878
BMI	−0.259	< 0.0001	−0.065	0.258	−0.076	0.174	−0.074	0.210	−0.044	0.426	−0.019	0.723
Scigarette smoking	−0.045	0.477										
Hypertension	−0.207	0.0008	−0.033	0.562	−0.042	0.448	−0.035	0.555	−0.024	0.656	−0.025	0.625
Dyslipidemia	−0.351	< 0.0001	−0.151	0.042	−0.189	0.009	−0.187	0.017	−0.162	0.023	−0.098	0.149
Diabetes mellitus	−0.138	0.025	0.056	0.433	0.068	0.331	0.056	0.445	0.023	0.733	−0.006	0.926
HbA1c	−0.154	0.013	−0.025	0.732	−0.037	0.598	−0.017	0.820	0.014	0.839	−0.001	0.987
HDL-C	0.301	< 0.0001	0.055	0.347	−0.002	0.969	0.131	0.026	0.213	0.0001	0.313	< 0.0001
Statins use	−0.193	0.002	0.005	0.937	−0.002	0.970	−0.038	0.583	0.007	0.917	−0.049	0.416
Fibrates use	−0.269	< 0.0001	−0.167	0.002	−0.147	0.005	−0.196	0.0003	−0.156	0.002	−0.109	0.025
Glitazone use	0.024	0.702										
TRLs-related markers												
TG*	−*0.601*	< *0.0001*	−*0.436*	< *0.0001*	–		–		–		–	
VLDL	−*0.606*	< *0.0001*	–		−*0.464*	< *0.0001*	–		–		–	
RLP-C*	−*0.502*	< *0.0001*	–		–		−*0.349*	< *0.0001*	–		–	
apo C-II	−*0.577*	< *0.0001*	–		–		–		−*0.453*	< *0.0001*	–	
apo C-III	−*0.591*	< *0.0001*	–		–		–		–		−*0.544*	< *0.0001*
Inflammatory markers												
WBC count	−0.182	0.003	−0.078	0.144	−0.079	0.134	−0.106	0.056	−0.097	0.058	−0.075	0.123
hs-CRP*	−0.083	0.184										
Lipid-modifying treatment (n = 335)												
Age	−0.138	0.012	−0.176	0.001	−0.170	0.001	−0.186	0.001	−0.222	< 0.0001	−0.231	< 0.0001
Gender	−0.201	0.0002	−0.176	0.002	−0.143	0.008	−0.162	0.006	−0.166	0.003	−0.131	0.014
BMI	0.004	0.939										
Scigarette smoking	−0.036	0.510										
Hypertension	−0.144	0.009	−0.075	0.155	−0.077	0.129	−0.077	0.164	−0.075	0.161	−0.054	0.281
Dyslipidemia	−0.120	0.029	−0.033	0.534	−0.042	0.406	−0.039	0.480	−0.028	0.604	−0.029	0.563
Diabetes mellitus	−0.094	0.089										
HbA1c	−0.116	0.039	−0.065	0.218	−0.056	0.268	−0.056	0.309	−0.033	0.540	−0.044	0.381
HDL-C	0.220	< 0.0001	0.027	0.635	−0.043	0.449	0.092	0.113	0.123	0.027	0.203	0.0002
Statins use	0.019	0.731										
Fibrates use	−0.132	0.016	−0.088	0.945	−0.072	0.153	−0.108	0.051	−0.092	0.083	−0.066	0.187
Glitazone use	0.014	0.800										
TRLs-related markers												
TG*	−*0.368*	< *0.0001*	−*0.299*	< *0.0001*	–		–		–		–	
VLDL	−*0.468*	< *0.0001*	–		−*0.424*	< *0.0001*	–		–		–	
RLP-C*	−*0.208*	*0.0002*	–		–		−*0.133*	*0.019*	–		–	
apo C-II	−*0.253*	< *0.0001*	–		–		–		−*0.249*	< *0.0001*	–	
apo C-III	−*0.384*	< *0.0001*	–		–		–		–		−*0.416*	< *0.0001*
Inflammatory markers												
WBC count	−0.114	0.037	−0.038	0.495	−0.040	0.441	−0.075	0.192	−0.060	0.278	−0.006	0.916
hs-CRP*	−0.060	0.276										
No Lipid-modifying treatment (n = 349)												
Age	−0.105	0.051										
Gender	−0.203	0.0002	−0.073	0.163	−0.063	0.218	−0.072	0.178	−0.036	0.282	−0.008	0.867
BMI	−0.213	< 0.0001	0.041	0.462	0.008	0.879	0.027	0.637	0.036	0.508	0.074	0.158
Scigarette smoking	−0.087	0.119										
Hypertension	−0.084	0.121										
Dyslipidemia	−0.153	0.005	0.002	0.965	−0.039	0.475	−0.009	0.88	0.020	0.725	0.070	0.190
Diabetes mellitus	−0.116	0.032	−0.03	0.575	0.006	0.916	−0.047	0.405	−0.040	0.451	−0.005	0.267
HbA1c	−0.078	0.153										
HDL−C	0.354	< 0.0001	0.187	0.002	0.112	0.067	0.259	< 0.0001	0.349	< 0.0001	0.480	< 0.0001
Glitazone use	−0.046	0.392										
TRLs-related markers												
TG*	−*0.490*	< *0.0001*	−*0.420*	< *0.0001*	–		–		–		–	
VLDL	−*0.518*	< *0.0001*	–		−*0.446*	< *0.0001*	–		–		–	
RLP-C*	−*0.408*	< *0.0001*	–		–		−*0.355*	< *0.0001*	–		–	
apo C-II	−*0.403*	< *0.0001*	–		–		–		−*0.417*	< *0.0001*	–	
apo C-III	−*0.410*	< *0.0001*	–		–		–		–		−*0.528*	< *0.0001*
Inflammatory markers												
WBC count	−0.140	0.010	−0.012	0.822	0.002	0.968	−0.018	0.736	−0.037	0.471	−0.030	0.528
hs-CRP*	−0.160	0.003	−0.035	0.511	0.006	0.916	−0.047	0.405	−0.037	0.471	−0.018	0.714

*BMI* body mass index, *eGFR* estimated glomelular flow rate; *CKD* chronic kidney disease, *CAD* coronary artery disease, *AP* angina pectoris, *OMI* old myocardial infarction, *Hb* hemoglobin, *ACE* angiotensin-converting enzyme, *ARB* angiotensin receptor blocker, *TC* total cholesterol, *LDL* low-density lipoprotein, *HDL* high-density lipoprotein, *TG* triglyceride, *VLDL* very LDL, *RLP* remnant-like particle, apo apolipoprotein, *MDA* malondealdehyde-modified, *WBC* white blood count, *hs-CRP* high-sensitivity C-reactive protein, *r* correlation coefficient, *β* standard partial regression coefficient, * log-transformed value was used; gender (0: female, 1: male); cigarette smoking (0: no, 1: yes); hypertension (0: no. 1: yes); diabetes mellitus (0: no, 1: yes); statins use (0: no. 1: yes); fibrates use (0: no. 1: yes); glitazones use (0: no. 1: yes)

**Table 5:** Correlations between the LDL-C/apo B ratio and TRLs-related markers among the 4 Groups

TRLs-related markers	CAD (+) DM (+) n = 60		CAD (+) DM (−) n = 90		CAD (−) DM (+) n = 118		CAD (−) DM (−) n = 416	
	r	p value	r	p value	r	p value	r	p value
TG	−0.351	0.007	−0.275	0.009	−0.62	< 0.0001	−0.49	< 0.0001
VLDL	−0.332	0.01	−0.314	0.003	−0.585	< 0.0001	−0.512	< 0.0001
RLP-C	−0.287	0.026	−0.175	0.107	−0.508	< 0.0001	−0.364	< 0.0001
apo C-II	−0.283	0.029	−0.281	0.008	−0.354	0.0001	−0.366	< 0.0001
apo C-III	−0.318	0.013	−0.356	0.001	−0.496	< 0.0001	−0.395	< 0.0001

*TC* total cholesterol, *LDL* low-density lipoprotein, *HDL* high-density lipoprotein, *TG* triglyceride, *VLDL* very LDL, *RLP* remnant-like particle, apo apolipoprotein, *MDA* malondealdehyde-modified, *WBC* white blood count, *hs-CRP* high-sensitivity C-reactive protein, *r* correlation coefficient

### Multi-logistic regression analysis to identify the variables that were independently correlated with changes of the LDL-C/apoB ratio

In this cross-sectional study, we confirmed that increased levels of TRL-related markers were associated with a decrease of the LDL-C/apoB ratio. Therefore, we investigated, using the longitudinal method, the relationship between the absolute changes (∆) in the serum TG levels and the ∆ LDL-C/apoB ratio, in order to examine the causal relationship. During a follow up period of at least 6 months, multivariable logistic regression analysis conducted in the 445 patients who were followed up for at least 6 months after adjustments for age, gender and risk factors for CAD revealed that higher ∆ serum TG was an independent predictor of a decreased LDL-C/apoB ratio. Next, similar analyses were performed in patients with serum LDL-C levels of < 100 mg/dL, and patients taking/not taking lipid-modifying treatments. Statistical analyses revealed similar findings (Table 6). Similarly, all the multi-logistic regression analyses showed that higher ∆ values of other TRL-related markers were independent determinants of a decreased ∆ LDL-C/apoB ratio (data not shown).

**Table 6:** Multi-logistic regression analysis to identify the variables that were independently correlated with changes of the LDL-C/apoB ratio

Variables	95% CI			
	OR	Upper	Lower	p value
All cases, n = 445				
⊿TG	1.006	1.003	1.009	< 0.0001
⊿FBG	0.999	0.993	1.005	0.749
⊿HbA1c	2.112	0.843	5.259	0.108
Age	0.988	0.971	1.006	0.186
Gender	0.836	0.547	1.277	0.407
BMI	0.982	0.929	1.037	0.503
Cigarette smoking	0.968	0.535	1.751	0.914
Hypertension	1.67	1.008	2.766	0.047
Lipid-modufying treatment	0.972	0.651	1.451	0.889
LDL-C < 100 mg/dL, n = 188				
⊿TG	1.007	1.002	1.011	0.004
⊿FBG	1.003	0.993	1.013	0.533
⊿ HbA1c	0.932	0.276	3.144	0.91
Age	0.991	0.962	1.021	0.563
Gender	0.963	0.469	1.978	0.918
BMI	0.877	0.897	1.066	0.604
Cigarette smoking	1.446	0.569	3.671	0.438
Hypertension	1.654	0.7	3.907	0.251
Lipid-modufying treatment	1.335	0.67	2.659	0.412
Lipid-modifying treatment, n = 270				
⊿TG	1.005	1.002	1.009	0.003
⊿FBG	0.998	0.991	1.005	0.56
⊿HbA1c	3.06	0.988	9.483	0.053
Age	0.979	0.954	1.006	0.121
Gender	0.825	0.479	1.423	0.489
BMI	0.979	0.911	1.052	0.564
Cigarette smoking	0.735	0.334	1.618	0.444
Hypertension	1.45	0.751	2.799	0.269
No lipid-modifying treatment, n = 175				
⊿TG	1.008	1.003	1.013	0.003
⊿FBG	1.002	0.991	1.013	0.73
⊿HbA1c	0.933	0.177	4.924	0.934
Age	0.998	0.975	1.022	0.876
Gender	0.817	0.403	1.022	0.876
BMI	0.989	0.903	1.082	0.805
Cigarette smoking	1.444	0.571	3.654	0.438
Hypertension	2.351	1.033	5.343	0.042

*BMI* body mass index, *eGFR* estimated glomelular flow rate; *CKD* chronic kidney disease, *CAD* coronary artery disease, *AP* angina pectoris, *OMI* old myocardial infarction, *Hb* hemoglobin, *ACE* angiotensin-converting enzyme, *ARB* angiotensin receptor blocker, *TC* total cholesterol, *LDL* low-density lipoprotein, *HDL* high-density lipoprotein, *TG* triglyceride, *VLDL* very LDL, *RLP* remnant-like particle, apo apolipoprotein, *MDA* malondealdehyde-modified, *WBC* white blood count, *hs-CRP* high-sensitivity C-reactive protein, *OR* odds ratio, *CI* confidence interval, ∆ absolute change from baseline, gender (0: female, 1: male); cigarette smoking (0: no, 1: yes); hypertension (0: no. 1: yes); lipid-modifying treatment (0: no. 1: yes)

## Discussion

In this study we showed that the LDL metabolism abnormality in CAD patients with DM is a pathological condition that strongly induces a decrease in LDL-particle size. A similar phenomenon occurs even in patients whose serum LDL-C levels are well controlled, and impaired TG metabolism plays a large role. Although this study evaluated the risk of CAD in DM by means of a cross-sectional and longitudinal design that focused on LDL-particle size and TG metabolism, the results may indicate the necessity of monitoring the qualitative changes in LDL-C, in addition to the quantitative changes, especially in CAD patients with DM.

Triglyceride is known as the most powerful determinant of the LDL-particle size [6]. LDL-particle size shows a significantly negative correlation with the fasting and postprandial serum TG levels, and is associated with postprandial hyperlipidemia often seen in patients with CAD and/or DM [14, 15]. Metabolism of TG-rich large VLDL is slower than that of ordinary VLDL. In cases where smaller LDL-particles are predominant, the formation of large VLDL increases, and accumulation of TRLs occurs under the influence of increased large VLDL formation and delayed catabolism of the large VLDL. Transfer of lipids takes place between the TRLs increased thus and HDL, leading to an increase of TG-rich LDL and formation of smaller LDL through degradation of TG via hepatic lipase activity [16]. As illustrated above, investigations have been reported concerning the association of increase in TRLs (as a result of abnormal TG metabolism) with reduction of the LDL-particle size. The results of the present study may be interpreted as indicating that promotion of LDL-particle size reduction by the above-mentioned abnormal TG metabolism is more marked in CAD patients with underlying DM.

In the multi-logistic regression analysis shown in Fig. 2, correction was made for an independent variable, i.e., the presence/absence of medication affecting the LDL-particle size. However, it is difficult to completely eliminate the influence of medication use on the LDL-particle size. Paradoxically, a tendency towards a lower LDL-particle size may not be avoidable in diabetic patients with CAD if the TG metabolism remains abnormal despite favorable blood glucose control (even in cases where favorable blood glucose control is accompanied by satisfactory serum LDL-C control with lipid metabolism-improving agents such as statins).

The Pioglitazone Effect on Regression of Intravascular Sonographic Coronary Obstruction Prospective Evaluation (PERISCOPE trial) [17], a randomized trial comparing glimepiride and pioglitazone that investigated the prevention of coronary plaque progression, reported not finding a significant difference in glycemic control between the two groups, but that the TG/HDL-C ratio, the ratio of a high TG level to a low HDL-C level that is a typical characteristic of lipid metabolism abnormality in DM [6], was significantly lower in the pioglitazone group, and that it contributed to preventing coronary plaque progression. Interestingly, it has also been reported that the TG/HDL-C ratio is a marker of abnormal TG metabolism and that the ratio is inversely correlated with LDL-particle size [18, 19]. This evidence appears to support our results. Thus, it is clear that glycemic and LDL-C control are important in preventing CV events in diabetic patients with CAD, and that improving abnormal TG metabolism may also be an important prevention strategy. It is necessary to regulate dysllipidemia in patients with DM. This can be done with lipid-lowering agents (e.g. statins, possibly in combination with a fibrate, niacin, omega-3 fatty acids, or ezetimibe) have proved effective in reducing atherogenic cholesterol particles including TRLs, inhibiting the progress of atherosclerosis [20].

In the present study, statistically significantly negative correlations were observed between the LDL-C/apoB ratio and most TRL-related markers in all the four groups. When this observation is considered with the results of multi-logistic regression analysis, we may say that the present study supported validity of our hypothesis that reduction of the LDL-particle size, which can be induced by abnormal TG metabolism, is more marked in CAD patients with underlying DM. If the above-mentioned results are considered with the finding of a higher TG/HDL-C ratio in the CAD patients with underlying DM than in the CAD patients without DM, we may say that the more advanced coronary atherosclerosis in CAD patients with underlying DM is the reason for the higher prevalence of more advanced cases or cases with complex lesions seen in this patient group.

As shown in Table 3, in the present study, we compared the estimated LDL-particle size (based on the TG/HDL-C ratio) in patients with/without CAD and DM. The results suggest that the TG/HDL-C ratio may also serve as a useful marker of the LDL-particle size. Thus, it seems necessary, in the future, to discuss which of LDL-C/apoB ratio and TG/HDL-C ratio should be selected as a marker of the LDL-particle size depending on the features of the study population or study design.

Interestingly, some investigations [21, 22] have reported that the LDL-C/apo B ratio is independently associated with the future development of cardiometabolic syndrome which involves characteristic lipid abnormalities such as hypertriglycemia and smaller LDL-particle size. Thus, the LDL-C/apo B ratio and TRLs may provide useful information when assessing atherosclerotic cardiovascular risks.

Furthermore, an additional study by a longitudinal method revealed that elevated levels of TRL-related markers were independently predictive of a decreased LDL-particle size. Due to its observational design, we were unable to establish a causal relationship in this study, but the results of the 2 studies with different (cross-sectional and longitudinal) designs taken together strongly suggest an association between increased levels of TRL-related markers and decrease of the LDL-particle size in patients with disordered TG metabolism; thus, the LDL-C/apoB ratio may serve as a useful predictor of the future development of CAD.

In the present study, we indirectly analyzed the association of the LDL-C/apoB ratio with the severity of coronary atherosclerosis in CAD patients with underlying DM. However, it is important to bear in mind that the progression of coronary atherosclerosis is powerfully stimulated by interactions among diabetes-associated factors (insulin resistance, abnormal glucose tolerance, etc.) and other factors such as abnormal lipid metabolism [5].

### Study limitations and clinical implications

First, the relationships bewteen the LDL-C/apoB ratio and TRL-related markers were analyzed by dividing the patients according to the history (positive/negative) of intake of lipid-modifying drug treatment and good serum LDL-C control, because lipid modifying drugs have an effect of improving the LDL and TG metabolism. The possibility of the effects of lipid modifying drugs influencing the results of the present study cannot be excluded. It had also been reported that the influence on the LDL-particle size varies among the different types of statins [23]. Furthermore, the duration of treatment involving such drugs could not be ascertained in the present study. Second, in theory the LDL-C/apoB ratio is a marker of a patient’s mean LDL-particle diameter, but it does not indicate the exact LDL-particle diameter, which is measured using density gradient ultracentrifugation and nuclear magnetic resonance spectroscopy. Moreover, the significance of calculating the absolute LDL-C/apoB ratio cut-off value for CAD risk has not been determined. Third, no patients who were taking dipeptidyl peptidase (DPP)-4 inhibitors or sodium-glucose transporter (SGLT) 2 inhibitors were included among the subjects of this study. DPP-4 inhibitors and SGLT2 inhibitors have triglyceride lowing actions, and it would be very interesting to evaluate these actions comparatively [24, 25]. Fourth, because determination of the presence of CAD in this study population relied on the findings of coronary angiography, the existence of subjects in the study population of undetected cases of asymptomatic CAD which can be diagnosed primarily by an exercise stress test or non-invasive tests such as coronary artery computed tomography cannot be ruled out. Diabetic patients often have asymptomatic myocardial ischemia, and a particularly high prevalence of asymptomatic myocardial ischemia has been reported in diabetic patients with CAD and abnormal TG metabolism [26]. Finally, in the future, an interventional study to investigate the causal relationship is needed.

## Conclusions

To further reduce the coronary risk in CAD patients with underlying DM, it may be of particular importance to pay attention not only to the quantitative changes of the serum LDL-C, but also to disorders of TG metabolism associated with LDL heterogeneity. Combined evaluation of TRL-related markers and the LDL-C/apoB ratio may be useful for assessing the risk status of CAD patients with underlying DM. Further studies are needed to investigate clinical outcomes of these patients.

## Abbreviations

ANOVA: analysis of variance
apo: apolipoprotein
CAD: coronary artery disease
CV: cardiovascular
DM: diabetes mellitus
DPP: dipeptidyl peptidase
eGFR: estimated glomerular filtration rate
Hb: hemoglobin
HDL: high-density lipoprotein
hs-CRP: high-sensitivity C-reactive protein
LDL-C: low-density lipoprotein cholesterol
MDA: malondealdehyde-modified
MDRD: Modification of Diet in Renal Disease
PERISCOPE: Pioglitazone Effect on Regression of Intravascular Sonographic Coronary Obstruction Prospective Evaluation
RLP: remnant-like particle
SD: standard deviation
Sd: small-dense
SGLT: sodium-glucose transporter
TC: total cholesterol
TG: triglyceride
TRL: TG-rich lipoprotein
VLDL: very-low density lipoprotein

## References

[1] Kannel WB, McGee DL (1979). Diabetes and cardiovascular disease. The Framingham study. JAMA.

[2] Mazzone T, Chait A, Plutzky J (2008). Cardiovascular disease risk in type 2 diabetes mellitus: insights from mechanistic studies. Lancet.

[3] Kennedy MW, Fabris E, Suryapranata H, Kedhi E (2017). Is ischemia the only factor predicting cardiovascular outcomes in all diabetes mellitus patients?. Cardiovasc Diabetol.

[4] Baigent C, Keech A, Kearney PM, Blackwell L, Buck G, Pollicino C (2005). Efficacy and safety of cholesterol-lowering treatment: prospective meta-analysis of data from 90,056 participants in 14 randomised trials of statins. Lancet.

[5] Han T, Cheng Y, Tian S, Wang L, Liang X, Duan W (2016). Changes in triglycerides and high-density lipoprotein cholesterol may precede peripheral insulin resistance, with 2-h insulin partially mediating this unidirectional relationship: a prospective cohort study. Cardiovasc Diabetol.

[6] Miller M, Stone NJ, Ballantyne C, Bittner V, Criqui MH, Ginsberg HN, American Heart Association Clinical Lipidology, Thrombosis, and Prevention Committee of the Council on Nutrition, Physical Activity, and Metabolism, Council on Arteriosclerosis, Thrombosis and Vascular Biology, Council on Cardiovascular Nursing, Council on the Kidney in Cardiovascular Disease (2011). Triglycerides and cardiovascular disease: a scientific statement from the American Heart Association. Circulation.

[7] Hirano T, Ito Y, Yoshino G (2005). Measurement of small dense low-density lipoprotein particles. J Atheroscler Thromb.

[8] Kaneva AM, Potolitsyna NN, Bojko ER (2017). Usefulness of the LDL-C/apoB ratio in the overall evaluation of atherogenicity of lipid profile. Arch Physiol Biochem.

[9] St-Pierre AC, Cantin B, Dagenais GR, Mauriège P, Bernard PM, Després JP (2005). Low-density lipoprotein subfractions and the long-term risk of ischemic heart disease in men: 13-year follow-up data from the Québec Cardiovascular Study. Arterioscler Thromb Vasc Biol.

[10] Tani S, Matsumoto M, Nagao K, Hirayama A (2014). Association of triglyceride-rich lipoproteins-related markers and low-density lipoprotein heterogeneity with cardiovascular risk: effectiveness of polyacrylamide-gel electrophoresis as a method of determining low-density lipoprotein particle size. J Cardiol.

[11] Sniderman AD, Blank D, Zakarian R, Bergeron J, Frohlich J (2003). Triglycerides and small dense LDL: the twin Achilles heels of the Friedewald formula. Clin Biochem.

[12] Matsuo S, Imai E, Horio M, Yasuda Y, Tomita K, Nitta K (2009). Collaborators developing the Japanese equation for estimated GFR: revised equations for estimated GFR from serum creatinine in Japan. Am J Kidney Dis.

[13] Gazi IF, Tsimihodimos V, Tselepis AD, Elisaf M, Mikhailidis DP (2007). Clinical importance and therapeutic modulation of small dense low-density lipoprotein particles. Expert Opin Biol Ther..

[14] Gong J, Fang K, Dong H, Wang D, Hu M, Lu F (2016). Effect of fenugreek on hyperglycaemia and hyperlipidemia in diabetes and prediabetes: a meta-analysis. J Ethnopharmacol.

[15] Borén J, Matikainen N, Adiels M, Taskinen MR (2014). Postprandial hypertriglyceridemia as a coronary risk factor. Clin Chim Acta.

[16] Packard CJ, Demant T, Stewart JP, Bedford D, Caslake MJ, Schwertfeger G (2000). Apolipoprotein B metabolism and the distribution of VLDL and LDL subfractions. J Lipid Res.

[17] Nicholls SJ, Tuzcu EM, Wolski K, Bayturan O, Lavoie A, Uno K (2011). Lowering the triglyceride/high-density lipoprotein cholesterol ratio is associated with the beneficial impact of pioglitazone on progression of coronary atherosclerosis in diabetic patients: insights from the PERISCOPE (Pioglitazone Effect on Regression of Intravascular Sonographic Coronary Obstruction Prospective Evaluation) study. J Am Coll Cardiol.

[18] Quispe R, Manalac RJ, Faridi KF, Blaha MJ, Toth PP, Kulkarni KR (2015). Relationship of the triglyceride to high-density lipoprotein cholesterol (TG/HDL-C) ratio to the remainder of the lipid profile: the Very Large Database of Lipids-4 (VLDL-4) study. Atherosclerosis..

[19] Maruyama C, Imamura K, Teramoto T (2003). Assessment of LDL particle size by triglyceride/HDL-cholesterol ratio in non-diabetic, healthy subjects without prominent hyperlipidemia. J Atheroscler Thromb..

[20] Rosenblit PD (2016). Common medications used by patients with type 2 diabetes mellitus: what are their effects on the lipid profile?. Cardiovasc Diabetol.

[21] Takagi H, Niwa M, Mizuno Y, Yamamoto H, Goto SN, Umemoto T (2014). Effects of rosuvastatin versus atorvastatin on small dense low-density lipoprotein: a meta-analysis of randomized trials. Heart Vessels.

[22] Kwon CH, Kim BJ, Kim BS, Kang JH (2011). Low-density lipoprotein cholesterol to apolipoprotein B ratio is independently associated with metabolic syndrome in Korean men. Metabolism.

[23] Onat A, Can G, Ciçek G, Ayhan E, Doğan Y (2010). Predictive value of serum apolipoprotein B/LDL-cholesterol ratio in cardiometabolic risk population-based cohort study. Clin Biochem.

[24] Tani S, Takahashi A, Nagao K, Hirayama A (2015). Effect of dipeptidyl peptidase-4 inhibitor, vildagliptin on plasminogen activator inhibitor-1 in patients with diabetes mellitus. Am J Cardiol.

[25] Zinman B, Wanner C, Lachin JM, Fitchett D, Bluhmki E, Hantel S (2015). EMPA-REG OUTCOME Investigators. Empagliflozin, Cardiovascular Outcomes, and Mortality in Type 2 Diabetes. N Engl J Med.

[26] Valensi P, Avignon A, Sultan A, Chanu B, Nguyen MT, Cosson E (2016). Atherogenic dyslipidemia and risk of silent coronary artery disease in asymptomatic patients with type 2 diabetes: a cross-sectional study. Cardiovasc Diabetol..

